# Identification of purity and prognosis‐related gene signature by network analysis and survival analysis in brain lower grade glioma

**DOI:** 10.1111/jcmm.15805

**Published:** 2020-08-31

**Authors:** Zujian Xiong, Yi Xiong, Hongwei Liu, Chang Li, Xuejun Li

**Affiliations:** ^1^ Department of Neurosurgery Xiangya Hospital Central South University Changsha P. R. China; ^2^ Xiangya School of Medicine Central South University Changsha P. R. China; ^3^ Hunan International Scientific and Technological Cooperation Base of Brain Tumor Research Xiangya Hospital Central South University Changsha P. R. China

## Abstract

Tumour microenvironment of brain lower grade glioma (LGG) consists of non‐tumour cells including stromal cells and immune cells mainly. These non‐tumour cells dilute the purity of LGG and play pivotal roles in tumour growth and development, thereby affecting patient prognosis. Tumour purity is also associated with molecular subtypes of LGG. In this study, we discovered the most relevant module to purity by weighted gene co‐expression network analysis (WGCNA) and afterwards performed consensus network analysis and survival analysis to filter 61 significant genes related to both purity and prognosis. In turn, we built a simplified model based on the calculation of purity score, and consensus measurement of purity estimation (CPE), with a satisfactory predictive performance by random forest regression. HLA‐E, MSN, GNG‐5, MYL12A, ITGB4, PDPN, AGTRAP, S100A4, PLSCR1, VAMP5 were selected as the most relevant genes correlating to both purity and prognosis. The risk score model based on the 10 genes could moderately predict patients’ overall survival. These 10 genes, respectively, were positively correlated positively to immunosuppressive cells like macrophage M2, but negatively correlated to patient prognosis, which may explain partially the poor prognosis with low‐purity group.

## 1 INTRODUCTION

Glioma is the most common and lethal tumour type in central nervous system (CNS). Compared to glioblastoma (GBM, WHO IV), lower grade glioma (LGG, WHO II, III) grow slowly with less malignancy. Because of bioinformatics study on gliomas, the WHO added molecular markers, such as IDH mutation status, chromosome 1p or 19q codeletion (1p/19q codel) status, into the glioma diagnostic guideline to improve the diagnosis and treatment accuracy.^1^ Apart from previous treatment on LGG like surgery, chemotherapy and radiotherapy, immunotherapy is now one of popular approaches deal with tumours. However, clinical outcome in large randomized clinical trials using checkpoint inhibitors was not satisfactory.^2^ The efficiency of immunotherapy partially influenced by tumour microenvironment which consists mainly of immune cells and stromal cells.^3^, ^4^ Thus, considering the non‐tumour components within the tumour is essential for improving therapy effect.

Tumour purity is defined as the proportion of tumour cells in tumour tissue. It used to be determined by pathologists through visual evaluation so that the results could be affected by the sensitivity of histopathology or interobserver bias.^5^ We adapted the consensus measurement of purity estimation (CPE) based on RNA‐seq data to evaluate the non‐tumour proportion within LGG and former studies elucidated that CPE shows good accuracy in purity evaluation.^6^, ^7^

## 2 METHODS

### 2.1 Data download and pre‐processing

This study collected 697 samples of LGG patient from TCGA and CGGA database. 516 samples’ RNA‐seq data and matched clinical data were acquired from TCGA database via Xena Browser developed by UCSC. 181 samples’ RNA‐seq data and clinical data were downloaded from CGGA database (http://www.cgga.org.cn).^8^ All RNA‐Seq data were normalized by the transcripts per kilobase million (TPM) method for further analysis.

### 2.2 Tumour purity estimate

Tumour purity scores were inferred by the CPE method as described previously.^6^ Please read the File [Link], [Link] for more details.

### 2.3 SCNA and mutation analysis

Somatic copy number alteration (SCNA) data were download form GDAV Firehose which were detected by GISTIC 2.0. MAF files of LGG were downloaded from TCGA database and performed by R/maftools.

### 2.4 Differentially expressed genes (DEGs) identification and functional enrichment analysis

Two purity groups were divided by X‐tile based on CPE value. DEGs were identified by R/limma. Gene ontology (GO) and pathway enrichment analysis (Kyoto Encyclopedia of Genes and Genomes (KEGG)) were performed by R/clusterProfiler.^9^ Gene set variation analysis (GSVA) was performed by R/GSVA, the reference gene sets were downloaded from MSigDB, and all parameters were set as advised.^10^ We used single‐sample gene set enrichment analysis (ssGSEA) to estimate the immune cellular fraction by R/GSVA and CIBERSORT.^11^

### 2.5 Survival analysis and model building

Survival‐related analysis was conducted by R/survival and R/survminer. We first perform WGCNA and consensus network analysis by R/WGCNA with calculated soft threshold to find the module most relevant to CPE with 200 times permutation, parameters were advised by (Figure S1D‐S1E).^12^ Then, target genes were filtered by log‐rank test and Pearson's correlation firstly and selected by Lasso regression and random forest algorism. We used risk score to build the model.

$$\text{Risk Score}=\sum_{i=1}^{N}\left(Expi*Coei\right)$$

*N*, *Expi* and *Coei* represented the number of signature genes, gene expression level and coefficient value, respectively. All TCGA patients were divided into training set (n = 344) and test set (n = 163) randomly to build the model and CGGA patients used as validation set. The receiver operating characteristic curve (ROC) was generated to assess the accuracy of the model.

### 2.6 Statistics Analysis

All statistical analyses were performed using R software, version 3.5.3. Continues variables between groups were compared by Student's t test, one‐way ANOVA with post hoc pairwise Bonferroni tests or the Wilcoxon rank‐sum test. Correlations between continuous variables were evaluated by Spearman or Pearson's correlation analysis. The method workflow was depicted by Figure S1A. For more method details, please see the File [Link], [Link].

## 3 RESULTS AND DISCUSSION

**FIGURE 1:**
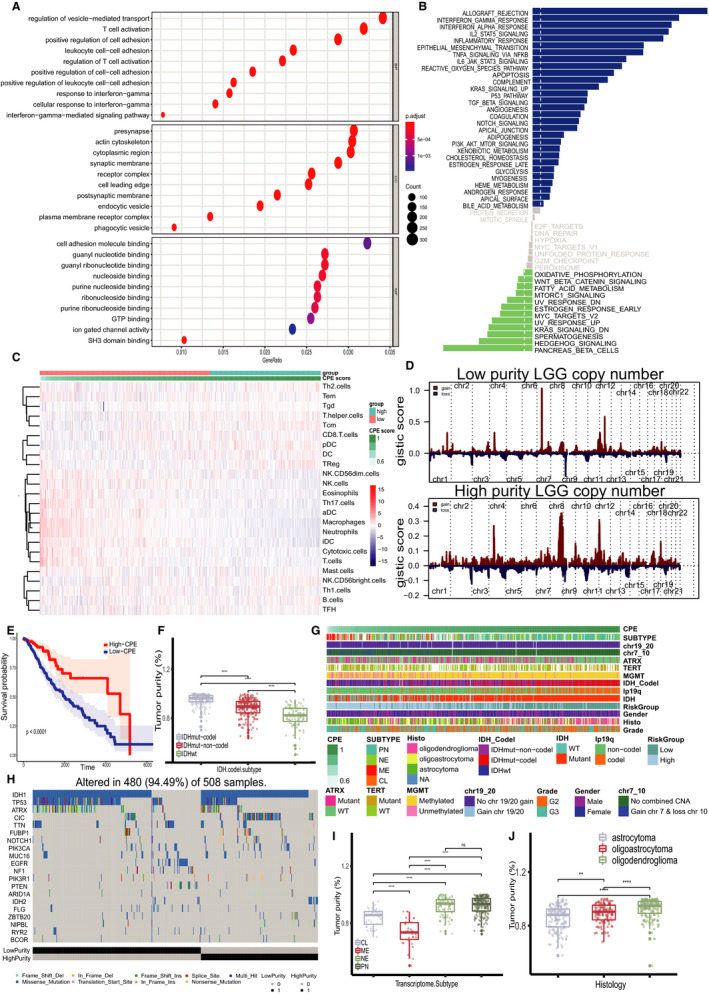
A, GO enrichment results of DEGs of low‐purity group comparing to high‐purity group. B, GSVA enrichment results of DEGs of low‐purity group comparing to high‐purity group. C, Immune infiltration evaluation by ssGSEA, the redder the immune cells are, the higher relative abundance the immune cells possess. D, CNV analysis results of low‐ and high‐purity group, CNV score were higher in low‐purity group with classical driver mutation of chromosome 7 gain and 10 loss. E, Kaplan‐Meier curve of high‐ and low‐purity groups, and low CPE means low purity indicated poor prognosis. F, The distribution of CPE value in different LGG molecular subtypes defined by WHO. IDHmut − codel, IDH mutation with 1p19q codeletion; IDHmut‐non‐codel, IDH mutation without 1p19q codeletion; IDHwt, IDH wild type. G, The relationship among LGG subtypes, specific molecular signature and purity score, CPE. H, Somatic variants of LGG between low‐ and high‐purity groups. I, The distribution of CPE in 4‐type classification. CL, classical subtype; ME, mesenchymal subtype; NE, neural subtype; PN, proneural subtype. J, Purity distribution in different histology of LGG

By calculating the DEGs between groups that divided by X‐tile based on CPE value, we found that these DEGs were enriched in immunological activity and immune cell‐cell adhesion pathways in low‐CPE group (Figure 1A‐B). However, we did not discover any metabolism pathway with statistical significance by KEGG. Hence, we assume that the type of biological metabolism of immune cells in the tumour microenvironment has little difference between groups. Meanwhile, the immune infiltration analysis revealed that more innate immune cells and tumour‐killing cells like macrophages, cytotoxic T cells and NK cells were accumulated in low‐purity tumour microenvironment, whereas adapted immune cells like T helper cells and B cells were more in high‐purity tumours, but the whole abundance of immune cells in high‐purity group was less than low‐purity group (Figure 1C). It was consistent with previous research that low purity accounts for positive immune status and intensive immune phenotype.^7^ On the other hand, the Kaplan‐Meier survival curve (Figure 1E) showed that low purity implied a poor prognosis with statistical significance. The high‐purity group possessed a better prognosis (median survival = 28.9 months) than the low‐purity group (median survival = 25.3 months). LGG tumour purity also correlated with molecular signatures such as IDH mutation, chromosome 1p/19q codeletion, chromosome 7 gain and 10 loss and the 4 LGG subtypes, classical, proneural, neural and mesenchymal subtypes (Figure 1G). The prognosis benefit mutation like IDH mutation and chromosome 1p19q codel were prone to exist in high‐purity group which could explain partially the better prognosis of high‐purity group (Figure 1F). Additionally, we also observed more classical driver mutations of glioma like chromosome 7 gain and 10 loss and mutation of TP53, EGFR and ATRX in low‐purity group which indicated the malignant potential of these tumours of low‐purity group (Figure 1D,1H). The histologic and molecular subtypes of LGG had different CPE value (Figure 1I‐J).

**FIGURE 2:**
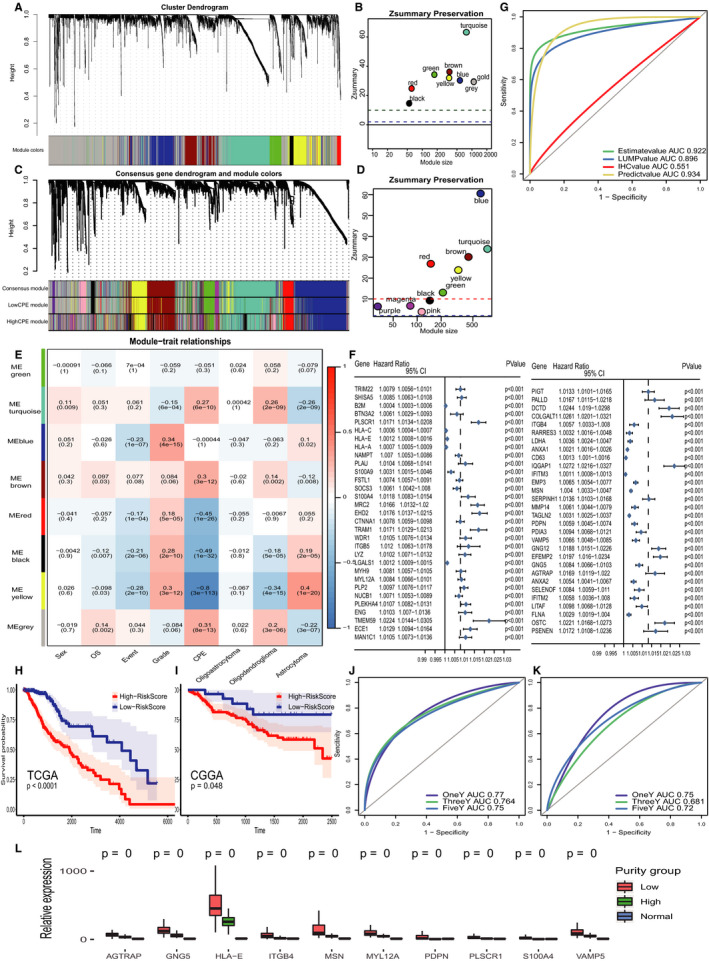
A, Identification of a co‐expression module in LGG by WGCNA. Red, black, yellow, gold, green, brown, blue and turquoise modules were identified, and grey module contains unmatched genes. B, Module preservation statistics of TCGA 8 modules and visualization, all modules were conservative due to Zsummary > 10. C, Identification of consensus modules between low‐ and high‐purity groups, based on consensus modules, low and high CPE groups had their own module organization respectively. D, Module preservation statistics of consensus network analysis and visualization, there were 4 unpreserved modules in low‐purity group comparing to high‐purity group, pink, magenta, purple and black. E, Correlation between the WGCNA co‐expression modules and clinical traits. The clinical traits included sex, overall survival (OS), dead event, grade, CPE and three glioma histological types: oligoastrocytoma, oligodendroglioma and astrocytoma. F, Forest plot of 61 filtered genes, all of which were identified as risk factors for patient prognosis. G, ROC curve of prediction efficiency of tumour purity. The constructed model had equivalent predictive value compared with ESTIMATE. H, Kaplan‐Meier curve of high‐ and low‐risk score group calculated by 10 genes in TCGA samples. I, Verification of risk score efficiency in CGGA samples’ prognosis. J, ROC curve of risk score prediction power on TCGA cohort. K, ROC curve of risk score prediction power on CGGA cohort. L, 10 genes expression in low‐purity group, high‐purity group and normal samples

Eight modules were identified by WGCNA, and all modules pass the preservation verification (Zsummary > 10) (Figure 2A‐B). It means all modules were conservative. Among them, the yellow module was identified to be the most correlated one with CPE value (MS = −0.8) and could distinguish the high‐ and low‐purity group well (Figure 2E, Figure S1B). We then used consensus network analysis to identify specific modules in the low‐purity group compared with the high‐purity group, and 10 modules were identified with 4 unconservative modules (Zsummary < 10) (Figure 2C‐D). We intersected these 4 modules with the former yellow module to get our 178 target genes, and these genes enriched at immune‐related pathways which means immune difference was the major difference between two groups (Figure S1C). We then conducted Pearson's correlation to CPE (coefficient > median), and log‐rank test (FDR < 0.05) on target genes, respectively, and then extracted the 85 common genes passed both tests. For further dimension reduction, we performed lasso regression to select 61 genes which also identified as risk factors by univariate Cox regression (Figure 2F). Purity prediction based on these 61 genes possessed similar power comparing to ESTIMATE that based on specific gene expression profiles of 141 immune genes and 141 stromal genes (Figure 2G) but with less genes. To simplify the model and connect purity with prognosis, we used 3 random forest algorism and selected 10 most correlative genes that ranked in the top 15 in all three algorisms and listed as follows: HLA‐E, MSN, GNG‐5, MYL12A, ITGB4, PDPN, AGTRAP, S100A4, PLSCR1, VAMP5 (Figure S2G–I, details of gene function and fold change please see the File [Link], [Link]). Comparing to normal samples, these 10 genes expression were higher in low‐purity group than high‐purity group than normal samples (Figure 2L). Additionally, we found that these 10 genes were positively correlated with PDCD1 with pretty good coefficients and moderately positively related to CTLA4 (Figure S2F). We then calculated each samples' risk score based on these 10 genes and divided TCGA samples into high‐ and low‐risk score groups with obvious prognostic difference, and risk score had moderately good prognostic prediction power (Figure 2H‐K). To validate risk score prediction power, we risk score into multiCox regression with gender, grade, IDH mutation, 1p19q codeletion and MGMT promoter methylation and the HR of risk score was 1.03 with FDR equalling 0.048. All these 10 genes were highly expressed in the high‐risk score group, associating with the low‐purity group in the TCGA data set and immune cell fractions. Also, we got consistent results in the CGGA dataset (Figure S2A‐D). Since innate immune cells dominated in the low‐purity LGG immune microenvironment, we observed less Treg cells infiltrated (Figure 1C). However, we observed that macrophage M2 fraction, which could promote tumour progression, metastasis and suppress anti‐tumour immune cells’ reactivity by expressing high levels of soluble factors such as TGF‐β, correlated positively with these 10 genes obviously, indicating high expression of these 10 genes correlated with high infiltration fraction of macrophage M2 (Figure S2E).^13^, ^14^ The increased innate immune suppressor infiltration could partially explain the poor prognosis caused by macrophage M2 facilitating tumour growth through induction of anti‐inflammatory responses and multitherapy resistance and impairing T cell activation and proliferation,^15^ and unsatisfactory outcome of checkpoint inhibitor that targeted to PD‐1, PD‐L1 or CTLA‐4 receptor, which usually expressed on T cell‐tumour cell interaction. This gave us a hint to pay attention on inhibiting innate anti‐immune cells like macrophage M2 to improve therapy outcomes. In summary, we identified the 10 most significant genes that contribute to both prognosis and purity. These genes showed a consistent correlation with different immune infiltrating cells between databases which could be further explored as potential targets in immune therapy.

## CONFLICT OF INTEREST

The authors state that the research was conducted without any commercial or financial relationship that could be interpreted as a potential conflict of interest.

## AUTHOR CONTRIBUTION

**Zujian Xiong:** Formal analysis (lead); Methodology (lead); Validation (lead); Visualization (lead); Writing‐original draft (lead); Writing‐review & editing (lead). **Yi Xiong:** Methodology (supporting); Validation (supporting); Visualization (supporting). **Hongwei Liu:** Methodology (supporting); Validation (supporting). **Chang Li:** Methodology (supporting); Writing‐review & editing (supporting). **Xuejun Li:** Conceptualization (lead); Funding acquisition (lead); Project administration (lead); Supervision (lead); Writing‐original draft (supporting); Writing‐review & editing (supporting).

## Supporting information

Figure S1

Figure S2

Table S1

Table S2

Supplementary Material

Supplementary Material

## References

[1] Louis DN , Perry A , Reifenberger G , et al. The 2016 world health organization classification of tumors of the central nervous system: a summary. Acta Neuropathol. 2016;131:803‐820. 27157931 10.1007/s00401-016-1545-1

[2] Mildenberger I , Bunse L , Ochs K , et al. The promises of immunotherapy in gliomas. Curr Opin Neurol. 2017;30:650‐658. 28984704 10.1097/WCO.0000000000000491

[3] Mirzaei R , Sarkar S , Yong VW . T Cell Exhaustion in glioblastoma: intricacies of immune checkpoints. Trends Immunol. 2017;38:104‐115. 27964820 10.1016/j.it.2016.11.005

[4] Golebiewska A , Bougnaud S , Stieber D , et al. Side population in human glioblastoma is non‐tumorigenic and characterizes brain endothelial cells. Brain : a journal of neurology. 2013;136:1462‐1475. 23460667 10.1093/brain/awt025 PMC3634193

[5] Cohen DA , Dabbs DJ , Cooper KL , et al. Interobserver agreement among pathologists for semiquantitative hormone receptor scoring in breast carcinoma. Am J Clin Pathol. 2012;138:796‐802. 23161712 10.1309/AJCP6DKRND5CKVDD

[6] Aran D , Sirota M , Butte AJ . Systematic pan‐cancer analysis of tumour purity. Nat Commun. 2015;6:8971. 26634437 10.1038/ncomms9971 PMC4671203

[7] Zhang C , Cheng W , Ren X , et al. Tumor purity as an underlying key factor in glioma. Clinical cancer research : an official journal of the American Association for Cancer Research. 2017;23:6279‐6291. 28754819 10.1158/1078-0432.CCR-16-2598

[8] Zhao Z , Meng F , Wang W , et al. Comprehensive RNA‐seq transcriptomic profiling in the malignant progression of gliomas. Scientific data. 2017;4:170024. 28291232 10.1038/sdata.2017.24 PMC5349247

[9] Yu G , Wang LG , Han Y , et al. clusterProfiler: an R package for comparing biological themes among gene clusters. OMICS. 2012;16:284‐287. 22455463 10.1089/omi.2011.0118 PMC3339379

[10] Hanzelmann S , Castelo R , Guinney J . GSVA: gene set variation analysis for microarray and RNA‐seq data. BMC Bioinformatics. 2013;14:7. 23323831 10.1186/1471-2105-14-7 PMC3618321

[11] Newman AM , Liu CL , Green MR , et al. Robust enumeration of cell subsets from tissue expression profiles. Nat Methods. 2015;12:453‐457. 25822800 10.1038/nmeth.3337 PMC4739640

[12] Langfelder P , Horvath S . WGCNA: an R package for weighted correlation network analysis. BMC Bioinformatics. 2008;9:559. 19114008 10.1186/1471-2105-9-559 PMC2631488

[13] Noy R , Pollard JW . Tumor‐associated macrophages: from mechanisms to therapy. Immunity. 2014;41:49‐61. 25035953 10.1016/j.immuni.2014.06.010 PMC4137410

[14] Binder H , Willscher E , Loeffler‐Wirth H , et al. DNA methylation, transcriptome and genetic copy number signatures of diffuse cerebral WHO grade II/III gliomas resolve cancer heterogeneity and development. Acta neuropathologica communications. 2019;7:59. 31023364 10.1186/s40478-019-0704-8 PMC6482573

[15] Ma Q , Long W , Xing C , et al. Cancer stem cells and immunosuppressive microenvironment in glioma. Front Immunol. 2018;9:2924. 30619286 10.3389/fimmu.2018.02924 PMC6308128

