# Supplementary figures and images for: Identification of purity and prognosis‐related gene signature by network analysis and survival analysis in brain lower grade glioma
Source: J Cell Mol Med. 2020 Aug 31;24(19):11607–12. doi: 10.1111/jcmm.15805 (PMC7576230; doi:10.1111/jcmm.15805)

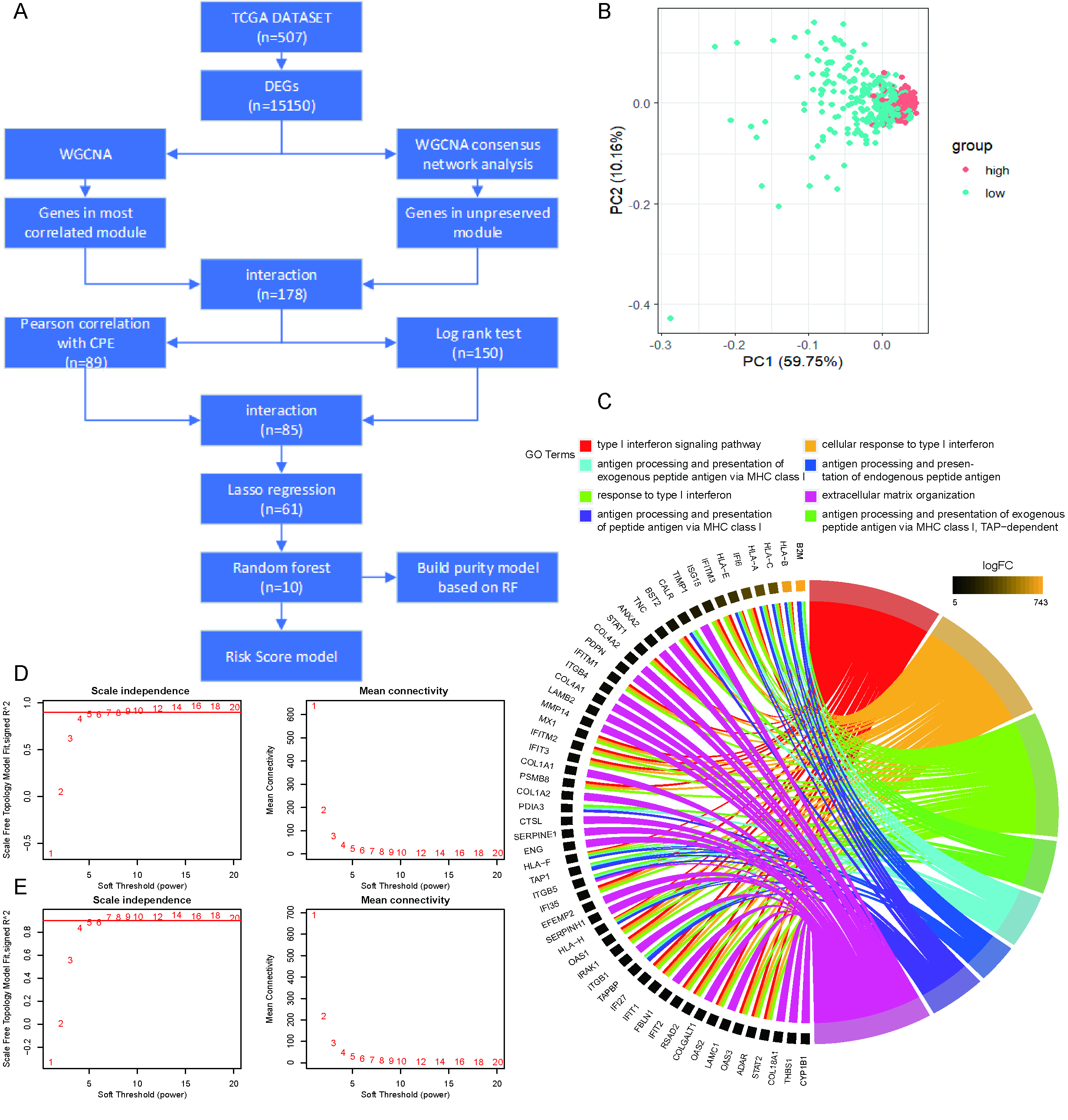

Supplement: Supplementary file 1 — Figure S1 [file JCMM-24-11607-s001.tif]

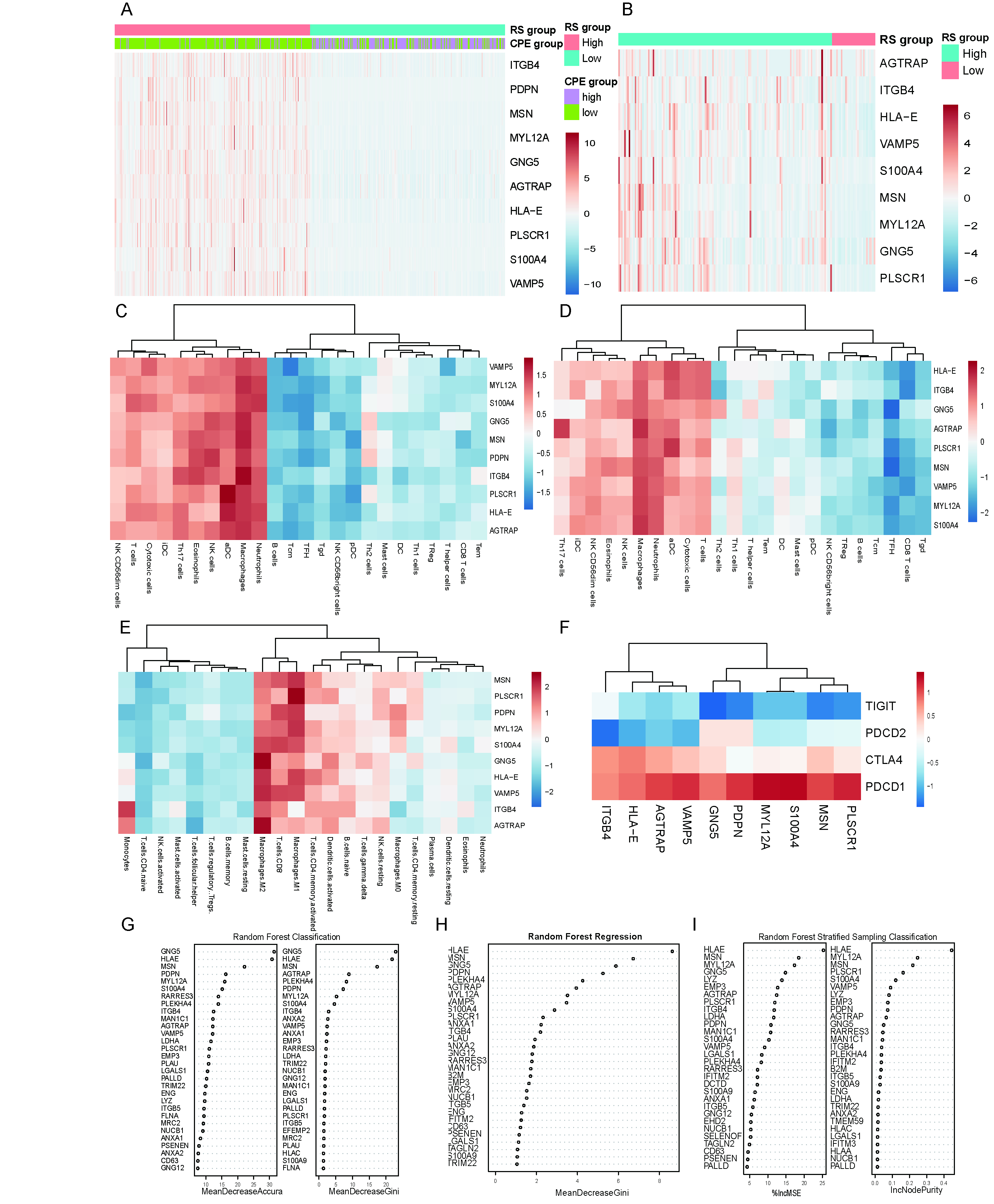

Supplement: Supplementary file 2 — Figure S2 [file JCMM-24-11607-s002.tif]
