# Supplementary material for: Identification of purity and prognosis‐related gene signature by network analysis and survival analysis in brain lower grade glioma
Source: J Cell Mol Med. 2020 Aug 31;24(19):11607–12. doi: 10.1111/jcmm.15805 (PMC7576230; doi:10.1111/jcmm.15805)
Supplement: Supplementary file 5 — Supplementary Material [file JCMM-24-11607-s005.docx]

**10 Gene Function and Fold Change**

|  | log2FC | | Function Role |
| --- | --- | --- | --- |
| HLA-E | | 275.5 | The only known ligand for CD94/NKG2A and CD94/NKG2C expressed on NK and CD8+ α-β and γ-δ T cells. It is mainly involved in maternal immune tolerance and may contribute to immunosuppression in glioma [1]. |
| MSN | | 113.2 | Related to the initiation and development of glioma [2]. High levels of MSN competitively displaced NF2 from CD44 and increase CD44 expression in a positive feedback loop driven by the Wnt/β-catenin signaling pathway, thereby activating various pathways and promoting tumor proliferation and metastasis [2]. Inhibiting MSN by miR-200c in glioma can inhibit tumor growth, invasion, and metastasis, and induce the occurrence of mesenchymal-epithelial-transitions (MET) [3] |
| S100A4 | | 20.1 | Neutrophils contribute to glioblastoma progression by supporting the expansion of the glioma stem cell pool in a manner that is dependent on S100 proteins, S100A4 specifically [4] |
| MYL12A | | 57.2 | A key player required to maintain the stability of myosin II and cellular integrity [5], and regulates cellular motility protein-protein interaction between myosin IIA and S100A4 [6] |
| ITGB4 | | 42.9 | A tumor-specific antigen, which is highly expressed in human glioma cells and is positively correlated with glioma grade. ITGB4 is also an important mediator of stem-like properties of glioma stem cells, contributing to glioma migration and proliferation in vivo and in vitro [7] |
| AGTRAP | | 33.5 | Serve as crucial modulators of the renin angiotensin system. It increases vascular density in the immature brain after hypoxic preconditioning [8]. But its function in glioma angiogenesis remains to be elucidated |
| PLSCR1 | | 23.2 | It upregulates in response to cytokines and other inflammatory stimuli [9] and can control the innate immune response intermediated by microglia. For instance, one report revealed that inhibiting its activity results in a nearly complete abrogation of microglia-mediated cell clearance in vivo and reduced microglial TAM receptor levels as well as TNF-α and IL-1β expression [10] |
| GNG-5 | | 74.0 | Involve in reducing E-cadherin expression through Wnt signaling pathway [11] |
| VAMP5 | | 57.8 | A component of synaptobrevin, serving the docking and fusion of vesicles and cell membranes |
| PDPN | | 46.4 | A transmembrane receptor glycoprotein that is upregulated on transformed cells, cancer-associated fibroblasts and inflammatory macrophages that contribute to cancer progression. Its high expression can increase the risk of venous thromboembolism in primary brain tumors via its ability to induce platelet aggregation [12] |

**Reference**

1. **Wischhusen J, Friese MA, Mittelbronn M*, et al.*** HLA-E protects glioma cells from NKG2D-mediated immune responses in vitro: implications for immune escape in vivo. *Journal of neuropathology and experimental neurology*. 2005; 64: 523-8.

2. **Zhu X, Morales FC, Agarwal NK*, et al.*** Moesin is a glioma progression marker that induces proliferation and Wnt/beta-catenin pathway activation via interaction with CD44. *Cancer research*. 2013; 73: 1142-55.

3. **Qin Y, Chen W, Liu B*, et al.*** MiR-200c Inhibits the Tumor Progression of Glioma via Targeting Moesin. *Theranostics*. 2017; 7: 1663-73.

4. **Liang J, Piao Y, Holmes L*, et al.*** Neutrophils promote the malignant glioma phenotype through S100A4. *Clinical cancer research : an official journal of the American Association for Cancer Research*. 2014; 20: 187-98.

5. **Park I, Han C, Jin S*, et al.*** Myosin regulatory light chains are required to maintain the stability of myosin II and cellular integrity. *The Biochemical journal*. 2011; 434: 171-80.

6. **Li ZH, Bresnick AR.** The S100A4 metastasis factor regulates cellular motility via a direct interaction with myosin-IIA. *Cancer research*. 2006; 66: 5173-80.

7. **Ma B, Zhang L, Zou Y*, et al.*** Reciprocal regulation of integrin β4 and KLF4 promotes gliomagenesis through maintaining cancer stem cell traits. *Journal of experimental & clinical cancer research : CR*. 2019; 38: 23.

8. **Gustavsson M, Mallard C, Vannucci SJ*, et al.*** Vascular response to hypoxic preconditioning in the immature brain. *Journal of cerebral blood flow and metabolism : official journal of the International Society of Cerebral Blood Flow and Metabolism*. 2007; 27: 928-38.

9. **Kodigepalli KM, Bowers K, Sharp A*, et al.*** Roles and regulation of phospholipid scramblases. *FEBS letters*. 2015; 589: 3-14.

10. **Tufail Y, Cook D, Fourgeaud L*, et al.*** Phosphatidylserine Exposure Controls Viral Innate Immune Responses by Microglia. *Neuron*. 2017; 93: 574-86.e8.

11. **Alsaleem M, Toss MS, Joseph C*, et al.*** The molecular mechanisms underlying reduced E-cadherin expression in invasive ductal carcinoma of the breast: high throughput analysis of large cohorts. *Modern pathology : an official journal of the United States and Canadian Academy of Pathology, Inc*. 2019.

12. **Riedl J, Preusser M, Nazari PM*, et al.*** Podoplanin expression in primary brain tumors induces platelet aggregation and increases risk of venous thromboembolism. *Blood*. 2017; 129: 1831-9.
