# Supplementary material for: Identification of purity and prognosis‐related gene signature by network analysis and survival analysis in brain lower grade glioma
Source: J Cell Mol Med. 2020 Aug 31;24(19):11607–12. doi: 10.1111/jcmm.15805 (PMC7576230; doi:10.1111/jcmm.15805)
Supplement: Supplementary file 6 — Supplementary Material [file JCMM-24-11607-s006.docx]

**Supplementary File -Methods**

**2.1 Data Download and Pre-processing**

This study collected 697 samples of human brain lower grade glioma (LGG) from 2 databases: CGGA (China) and TCGA (the United States). 516 samples of level 3 RNA-Seq data of LGG and normal sample data and matched clinical trait data were downloaded from the TCGA database (https://tcga-data.nci.nih.gov/) via Xena Browser developed by UCSC. 181 samples of RNA-Seq data and clinical trait data were collected from the CGGA database (<http://www.cgga.org.cn>). All RNA-Seq data were normalized by the Transcripts Per Kilobase Million (TPM) method for further analysis.

**2.2 Tumor Purity Analysis**

Tumor purity scores were inferred by the consensus purity estimation (CPE) method as previously described [1]. The tumor purity score was derived from the median value estimated from ESTIMATE, ABSOLUTE, LUMP and IHC methods after normalization. ESTIMATE is based on specific gene expression profiles of 141 immune genes and 141 stromal genes [2]; ABSOLUTE uses somatic copy-number data [3]; LUMP (leukocytes unmethylation for purity) averages 44 non-methylated immune-specific CpG sites and IHC is estimated by image analysis of hematoxylin and eosin stain slides produced by the Nationwide Children’s Hospital Biospecimen Core Resource [1]. CPE uses the median purity level of the normalized purity levels derived from the four methods mentioned above. Patients were divided into two groups according to the tumor purity scores. Differences in the clinical and molecular parameters, patient prognosis and transcriptome profiling between the high and low groups were further analyzed.

**2.3 SCNA and mutation analysis**

Somatic copy number alterations (SCNA) data (minus germline CNV) were downloaded from GDAC Firehose and separated into different datasets according to the purity. SCNA events were detected by GISTIC2.0 using the segmented Affymetrix SNP 6.0 microarray data [4]. Somatic variants files of LGGs in MAF format were downloaded from https ://tcga-data.nci.nih.gov/ via Xena Browser developed by UCSC and downstream analysis was performed by R/maftools package [5].

**2.4** **Differential Expressed Genes (DEGs) Identification and Functional Enrichment Analysis**

Two purity groups were generated by X-tile based on the CPE score, which is a graphical method that illustrates the presence of substantial tumor subpopulations and shows the robustness of the relationship between a biomarker and outcome by the construction of a two-dimensional projection of every possible subpopulation [6]. DEGs were identified by R package *limma* [7]. Gene ontology (GO) and pathway enrichment analysis (Kyoto Encyclopedia of Genes and Genomes (KEGG)) were performed using the R package clusterProfiler (https:// guangchuangyu.github.io/clusterProfiler) [8]. Enriched ontological terms and pathways with P < 0.05 were selected. Gene set variation analysis (GSVA) was performed by R package *GSVA,* the reference gene sets were downloaded from MSigDB and all parameters were set as advised [9].

**2.5 Survival Analysis and Model Building**

The Kaplan-Meier survival curve was depicted to estimate survival distribution by R packages named *survival* and *survminer*. The principal components analysis (PCA) package of R was used to profile transcriptomic patterns attributing to glioma purity. The log-rank test was used to assess statistical significance between groups to select survive related genes. Univariate Cox regression analyses were performed to identify independent prognostic genes. Lasso regression was performed by R package *lar*s. All patients of TCGA were randomly assigned to a training set (n = 344) (two-thirds for training model) and a testing set (n =163) (one-third for validating the prediction efficacy of model). In order to avoid leakage, we solely used the training set for model building. The model based on filtered genes was built by Random forest classification and regression respectively using R package *randomForest*. The receiver operating characteristic curve (ROC) was generated to assess the accuracy of the model with the R package *pROC* [10]. Genes weighted value was calculated based on a linear combination of the Cox coefficient and gene expression.

$$Risk Score=\sum_{i=1}^{N} (Expi*Coei)$$

N, Expi, and Coei represented the number of signature genes, gene expression level, and coefficient value, respectively. The formula was used to calculate a risk score, and a higher risk score predicts a worse prognosis. The cutoff value of high and low risk of the TCGA and CGGA cohorts was set as the median of the TCGA cohort.

**2.6 Weighted Gene Co-expression Network Analysis and Module Preservation**

WGCNA was performed using the R package *WGCNA* [11]. First, we filtered and reduced outliers of RNA-Seq samples. The co-expression similarity matrix consisted of the absolute values of the correlation between transcript expression levels. For an unsigned network, the correlation coefficient between gene i and j was defined as Sij: Sij = |cor(i,j)|. The co-expression similarity matrix of 3,690 genes with the highest expression variance (10% of the whole genes) was transformed into an adjacency matrix by choosing 9 as a soft threshold (Fig. S1D). The topology matrix was created using topological overlap measurement (TOM) - an advanced co-expression measurement method that takes into account not only the interrelationships between the two gene but also the extent to which they share correlations in a weighted network. Finally, we chose the dynamic hybrid cut method, a bottom-up algorithm, to identify co-expression gene modules based on their topological overlap matrix [12]. To determine the importance of each module, we calculated gene significance (GS) to measure the correlation between the gene and the clinical characteristics of the sample. Module significance (MS) is defined as the mean GS within the module and is calculated for correlation between the measurement module and the clinical characteristics of the sample (gender, OS, event, grade, CPE, Oligoastrocytoma, Oligodendroglioma, Astrocytoma) [13]. Statistical significance was determined using the correlation P-value. The module Preservation function in the *WGCNA* R package was used to calculate the Z summary to evaluate whether a module was conserved (nPermutations=200) [14].

After using X-tile [6] to divide TCGA samples into two groups based on CPE, consensus network analysis of WGCNA was performed between different CPE groups by the function *blockwiseConsensusModules* (power=7) (Fig. S1E) in *WGCNA* package constructing consensus modules, which was defined by linking two nodes if, and only if, a link exists in each of the input networks. The module Preservation function, *modulePreservation*, in the *WGCNA* R package was used to calculate the Z summary to evaluate whether a module was conserved (nPermutations=200).

**2.7 Immune Cellular Fraction Estimates**

The relative fraction of 24 immune cell types within the leukocyte compartment was estimated using gene sets introduced by Bindea et al.[15]. Immune cell types included both innate immune cells (dendritic cells (DCs), immature DCs (iDCs), activated DCs (aDCs), eosinophils, mast cells, macrophages, natural killer cells (NKs), NK CD56dim cells, NK CD56bright cells, and neutrophils) and adaptive immune cells (B, T helper 1 (Th1), Th2, T γ δ (Tgd), CD8+ T, T central memory (Tcm), T effector memory (Tem), and T follicular helper (Tfh) cells). RNA-Seq data was normalized and utilized as the input for immune infiltrating estimates and read count values for each gene were transformed into TPM. The immune cellular fraction was scored using the single-sample gene set enrichment analysis (ssGSEA) [16], as implemented in the *GSVA* R package[9]. The estimation of immune cell proportions for each sample was also performed by CIBERSORT[17] algorithm on TCGA and CGGA RNA-Seq data using LM22 as a reference expression signature with 200 permutations.

**2.8 Visualization**

Heatmaps were drawn by R package named *pheatmap*. Dot plots, bar plots and scatter plots were generated by *ggplot2*. The chord plot of GO results was visualized by *GOplot* [18]. Forest plots of selected genes were visualized by *forestplot*.

**2.9 Statistics Analysis**

All statistical analyses were performed using R software, version 3.5.3 (The R Foundation for Statistical Computing, http://www.rproject.org/). Continues variables between groups were compared by the Student’s t-test, one-way ANOVA with post hoc pairwise Bonferroni tests or the Wilcoxon rank-sum test. Pearson’s chi‐square tests were performed for the comparison of categorical variables. Correlations between continuous variables were evaluated by Spearman or Pearson correlation analysis.

**Reference**

1. **Aran D, Sirota M, Butte AJ.** Systematic pan-cancer analysis of tumour purity. *Nature communications*. 2015; 6: 8971.

2. **Yoshihara K, Shahmoradgoli M, Martinez E*, et al.*** Inferring tumour purity and stromal and immune cell admixture from expression data. *Nature communications*. 2013; 4: 2612.

3. **Carter SL, Cibulskis K, Helman E*, et al.*** Absolute quantification of somatic DNA alterations in human cancer. *Nature biotechnology*. 2012; 30: 413-21.

4. **Mermel CH, Schumacher SE, Hill B*, et al.*** GISTIC2.0 facilitates sensitive and confident localization of the targets of focal somatic copy-number alteration in human cancers. *Genome biology*. 2011; 12: R41.

5. **Mayakonda A, Lin DC, Assenov Y*, et al.*** Maftools: efficient and comprehensive analysis of somatic variants in cancer. *Genome research*. 2018; 28: 1747-56.

6. **Camp RL, Dolled-Filhart M, Rimm DL.** X-tile: a new bio-informatics tool for biomarker assessment and outcome-based cut-point optimization. *Clinical cancer research : an official journal of the American Association for Cancer Research*. 2004; 10: 7252-9.

7. **Ritchie ME, Phipson B, Wu D*, et al.*** limma powers differential expression analyses for RNA-sequencing and microarray studies. *Nucleic acids research*. 2015; 43: e47.

8. **Yu G, Wang LG, Han Y*, et al.*** clusterProfiler: an R package for comparing biological themes among gene clusters. *Omics : a journal of integrative biology*. 2012; 16: 284-7.

9. **Hanzelmann S, Castelo R, Guinney J.** GSVA: gene set variation analysis for microarray and RNA-seq data. *BMC bioinformatics*. 2013; 14: 7.

10. **Robin X, Turck N, Hainard A*, et al.*** pROC: an open-source package for R and S+ to analyze and compare ROC curves. *BMC bioinformatics*. 2011; 12: 77.

11. **Langfelder P, Horvath S.** WGCNA: an R package for weighted correlation network analysis. *BMC bioinformatics*. 2008; 9: 559.

12. **Langfelder P, Zhang B, Horvath S.** Defining clusters from a hierarchical cluster tree: the Dynamic Tree Cut package for R. *Bioinformatics (Oxford, England)*. 2008; 24: 719-20.

13. **Ghazalpour A, Doss S, Zhang B*, et al.*** Integrating genetic and network analysis to characterize genes related to mouse weight. *PLoS genetics*. 2006; 2: e130.

14. **Langfelder P, Luo R, Oldham MC*, et al.*** Is my network module preserved and reproducible? *PLoS computational biology*. 2011; 7: e1001057.

15. **Bindea G, Mlecnik B, Tosolini M*, et al.*** Spatiotemporal dynamics of intratumoral immune cells reveal the immune landscape in human cancer. *Immunity*. 2013; 39: 782-95.

16. **Barbie DA, Tamayo P, Boehm JS*, et al.*** Systematic RNA interference reveals that oncogenic KRAS-driven cancers require TBK1. *Nature*. 2009; 462: 108-12.

17. **Newman AM, Liu CL, Green MR*, et al.*** Robust enumeration of cell subsets from tissue expression profiles. *Nature methods*. 2015; 12: 453-7.

18. **Walter W, Sanchez-Cabo F, Ricote M.** GOplot: an R package for visually combining expression data with functional analysis. *Bioinformatics (Oxford, England)*. 2015; 31: 2912-4.
